# Prolactin, cortisol, and extracellular osmolality regulate *cftr*, *ostf1*, and *sgk1* in tilapia ionocytes

**DOI:** 10.3389/fendo.2026.1802254

**Published:** 2026-04-22

**Authors:** Jason P. Breves, Ming-Wen Hu, Mayu Inokuchi, Lucia A. Seale, Andre P. Seale

**Affiliations:** 1 Department of Biology, Skidmore College, Saratoga Springs, NY, United States; 2 Department of Ophthalmology, Johns Hopkins University School of Medicine, Baltimore, MD, United States; 3 Department of Aquatic Bioscience, Graduate School of Agricultural and Life Sciences, The University of Tokyo, Bunkyo, Tokyo, Japan; 4 Pacific Biosciences Research Center, University of Hawai’i at Mānoa, Honolulu, HI, United States; 5 Department of Human Nutrition, Food, and Animal Sciences, University of Hawai’i at Mānoa, Honolulu, HI, United States

**Keywords:** euryhaline, fish, gill, hormone, ion transporter, osmoregulation, RNA-Seq, salinity

## Abstract

**Objective:**

Euryhaline fishes can inhabit salinities ranging from fresh water (FW) to seawater (SW), in part because their endocrine system aligns the ion-transporting capacities of branchial ionocytes with the external environment. Ionocytes also directly sense extracellular osmolality and adjust their functions accordingly; therefore, this study investigated the interplay between osmotic conditions and osmoregulatory hormones in Mozambique tilapia (*Oreochromis mossambicus*) to further elucidate the basis of euryhalinity. We sought to determine whether prolactin (Prl) supports FW acclimation by counteracting osmotic and hormonal signals that initiate branchial responses to SW environments.

**Methods:**

We first combined hypophysectomy, hormone replacement, RNA-Seq, and qPCR to identify *cystic fibrosis transmembrane conductance regulator (cftr)*, *osmotic stress transcription factor 1 (ostf1)*, and *serum- and glucocorticoid-inducible kinase 1 (sgk1)* as Prl-repressed genes. We then leveraged a series of *in vivo* and *in vitro* experimental paradigms to characterize their regulation by environmental salinity, Prl, cortisol, and extracellular osmolality.

**Results:**

Our findings indicate that 1) Prl’s capacity to antagonize cortisol-stimulated *cftr* expression depends on extracellular osmotic conditions; 2) Prl and cortisol play opposing roles in regulating branchial *ostf1* expression; 3) Sgk1 is expressed in ‘SW-type’ ionocytes, where interactions among Prl, cortisol, and osmotic conditions influence its expression; and 4) Prl promotes the expression of *Na^+^/Cl^-^ cotransporter 2* and *Clc family Cl^−^ channel 2c*, as shown previously.

**Conclusions:**

The combined actions of osmotic stimuli, Prl, and cortisol shape the branchial expression of *cftr*, *ostf1*, and *sgk1*. While hyperosmotic extracellular conditions and cortisol promote the activation of these genes during SW acclimation, Prl supports FW adaptation by suppressing these genes and promoting processes underlying active ion uptake. Thus, euryhaline tilapia adjust the ion-transporting activity of their branchial ionocytes to meet environmental demands by integrating multiple regulatory cues.

## 1 Introduction

For teleost fishes to survive in marine environments, they must overcome physiological challenges posed by the chemical composition of seawater (SW). For instance, maintaining hydromineral balance requires expelling excess Na^+^ and Cl^-^ acquired from the environment. Through the activities of specialized ‘SW-type’ ionocytes, the branchial epithelium serves as the primary site for actively secreting Na^+^ and Cl^-^ into the surrounding salt-rich environment (1). In the basolateral membrane of ‘SW-type’ ionocytes, Na^+^/K^+^-ATPase (Nka) and Na^+^/K^+^/2Cl^-^ cotransporter 1 (Nkcc1) energize and facilitate the entry of Cl^-^ from the blood plasma into the cell (2). Apically expressed cystic fibrosis transmembrane conductance regulator (Cftr) permits the subsequent passage of Cl^-^ into the external environment (3). With Nkcc1 and Cftr providing the transcellular pathway for Cl^-^, Na^+^ exits the gill paracellularly by passing through tight junctions between ionocytes and neighboring accessory cells (4). This general paradigm for branchial Na^+^ and Cl^-^ secretion operates across teleosts (5). Therefore, when euryhaline species transition from freshwater (FW) to marine habitats, the activation of ‘SW-type’ ionocytes corresponds with increased *cftr*, *nkcc1*, and *nka-α1b* gene expression (6–10).

Conversely, when euryhaline fish transition from SW to FW, ion secretion must be swiftly attenuated to limit the deleterious loss of Na^+^ and Cl^-^. This is partially achieved through the downregulation of branchial *cftr* and *nkcc1* (7, 11, 12). Acclimation to FW also requires the activation of specialized ‘FW-type’ ionocytes that leverage a suite of ion transporters and channels to absorb environmental Na^+^ and Cl^-^. These transporters include Na^+^/Cl^-^ cotransporter 2 (Ncc2), Na^+^/H^+^ exchangers (Nhes), Cl^-^/HCO_3_^-^ exchangers, Na^+^/HCO_3_^-^ exchangers, and Clc family Cl^−^ channels (Clcs) (13, 14). Interestingly, ‘FW-type’ ionocytes do not operate in a generalizable manner across teleosts, and ionocyte-based strategies for Na^+^ and Cl^-^ uptake vary among species (14, 15).

In euryhaline species, the endocrine system plays a crucial role in aligning the ion-transporting capacities of ionocytes with environmental conditions (16). Among the many hormones that regulate ionocytes (17), prolactin (Prl) and cortisol are well-established regulators of ‘FW-’ and ‘SW-type’ ionocytes, respectively (18). For example, Prl promotes Na^+^ uptake, the recruitment of ‘FW-type’ ionocytes, and the transcription of *ncc2*, *nhe3*, *clc2c*, and *nka-α1a* (19–25). In teleosts, Prl signaling is mediated by two Prl receptors (Prlrs), Prlr1 and Prlr2, which are located in key osmoregulatory organs and differ in their transcriptional responses to salinity changes (26–28). Branchial *prlr1* expression increases when fish acclimate to FW (29), while *prlr2* expression increases when fish acclimate to SW (26). Alternatively, cortisol triggers adaptive responses to SW by stimulating branchial Nka activity along with *cftr* and *nkcc1* expression (7, 30–32). Although traditionally viewed as a ‘SW-adapting’ hormone, cortisol also promotes branchial ion-uptake pathways in particular species (18, 33).

In addition to systemic hormones, extracellular osmotic conditions directly influence ionocytes (22, 34–37). Because they interface with the external environment, ionocytes are subjected to changes in environmental osmolality at their apical surface. Additionally, deviations from internal osmotic set points during the acute phases of FW- and SW-acclimation can be sensed at their basolateral surface. Osmotic stress signaling utilizes transcription factors, kinases, and other intracellular signals to transduce osmotic stimuli, such as cell volume and cytoskeletal changes, into adaptive responses (38–41). For example, hyperosmotic stress is linked to gene transcription through osmotic stress transcription factor 1 (Ostf1) and to the trafficking of Cftr to the apical membrane of ‘SW-type’ ionocytes via serum- and glucocorticoid-inducible kinase 1 (Sgk1) (42–44). Since hormones and osmotic stress signals can act synergistically or antagonistically to modulate ion-transporter expression (22, 24), ionocytes must integrate hormonal cues with osmotic information to render adaptive responses to changes in salinity.

To investigate the interplay between endocrine axes and intracellular osmotic stress networks in fishes, we examined whether Prl counteracts factors that mediate branchial responses to hyperosmotic/SW environments. Drawing on the current understanding of how their branchial ionocytes, endocrine system, and osmotic stress signaling networks function, we selected the highly euryhaline Mozambique tilapia (*Oreochromis mossambicus*) for this study (45). Our *a priori* hypothesis was that Prl signaling downregulates *cftr* expression. In addition to exploring a connection between Prl and *cftr*, we sought to uncover Prl-repressed genes through the transcriptomic analysis of Prl-treated, hypophysectomized tilapia. After identifying *ostf1* and *sgk1* as Prl-repressed transcripts, we subsequently employed a series of *in vivo* and *in vitro* experimental paradigms to characterize their hormonal and osmotic regulation alongside *cftr*.

## 2 Materials and methods

### 2.1 Experimental animals and rearing conditions

Mozambique tilapia were obtained from a population maintained at the Hawai’i Institute of Marine Biology, University of Hawai’i. Fish were reared in outdoor tanks with a continuous flow of FW (municipal water; < 1‰) or SW (Kaneohe Bay, HI; 34‰) at 24-26 °C under natural photoperiod. Fish were fed twice daily with Trout Chow (Skretting, Tooele, UT). Gill filaments for immunohistochemical analyses were collected from male tilapia housed at the Skidmore College Animal Care Facility. Fish were maintained in recirculating artificial SW (Instant Ocean, Blacksburg, VA; 35‰) with particle and charcoal filtration at 23-25 °C under a 12L:12D light cycle. Fish were fed twice daily with Omega One cichlid pellets (Omega Sea, Painesville, OH). The University of Hawai’i and Skidmore College Institutional Animal Care and Use Committees approved all housing, surgical, and experimental procedures.

### 2.2 Hypophysectomy and Prl replacement

Hypophysectomy of male tilapia (80–100 g) was performed using the transorbital technique developed by Nishioka (46) and further described by Breves et al. (20). After a 5-day post-surgical recovery period in brackish water (BW; 12‰), hypophysectomized fish were administered ovine Prl (oPrl; 5 μg/g body weight, Sigma-Aldrich, St. Louis, MO) or vehicle (0.9% NaCl; 1.0 μl/g body weight) (*n* = 8) via intraperitoneal injections over 5 days. The oPrl dose was selected based on previous studies in tilapias (*Oreochromis* sp.), mummichogs (*Fundulus heteroclitus)*, channel catfish (*Ictalurus punctatus*), zebrafish (*Danio rerio*), and other teleosts, in which oPrl was injected intraperitoneally (21, 47–55). Our previous study established that *prlr1* and *prlr2* remain highly expressed in the gill following hypophysectomy (20). Forty-eight hours after the initial injection, the second and third injections were administered 48 h apart. Twenty-four hours after the third injection, all fish were anesthetized with 2-phenoxyethanol (2-PE; 0.3 ml/l, Sigma-Aldrich), and gill filaments were excised from the first arch (left side) and stored in TRI Reagent (MRC, Cincinnati, OH). Blood was collected from the caudal vasculature using a needle and syringe treated with heparin ammonium salt (200 U/mL, Sigma-Aldrich). Plasma was separated by centrifugation for plasma osmolality measurements using a vapor pressure osmometer (Wescor 5100C, Logan, UT). Fish were not fed during the recovery and post-injection periods. The completeness of hypophysectomy was confirmed by post-mortem inspection of the hypothalamic region. We note that hypophysectomy inherently impacts all pituitary hormones; therefore, caution should be taken when evaluating the extent of Prl’s actions, as potentially critical pituitary-based or pituitary-controlled intermediaries may be absent or disrupted.

### 2.3 RNA extraction and transcriptomic next-generation sequencing

To identify genes downregulated by Prl, we identified differentially expressed genes (DEGs) through RNA-Seq analysis of a subset of individuals (3 fish/treatment) from the hypophysectomy/replacement experiment. Total RNA was extracted from homogenized filaments using TRI Reagent according to the manufacturer’s protocol. RNA concentration and purity (1.9 < A260/A280 < 2.2) were initially assessed using spectrophotometric absorbance (NanoDrop One, ThermoFisher, Waltham, MA). RNA library preparation, sequencing, data quality control, and genome alignment were conducted at Azenta Life Sciences (South Plainfield, NJ). The workflow is briefly summarized below.

Total RNA samples were quantified using a Qubit 2.0 Fluorometer (Life Technologies, Carlsbad, CA), and RNA integrity was checked using the Agilent TapeStation 4200 (Agilent Technologies, Palo Alto, CA). RNA sequencing libraries were prepared using a NEBNext Ultra II RNA Library Prep Kit for Illumina (New England Biolabs, Ipswich, MA). The sequencing libraries were subsequently multiplexed and clustered onto an Illumina NovaSeq instrument flow cell. The samples were sequenced using a 2x150 bp paired-end configuration. NovaSeq Control Software conducted image analysis and base calling. Raw sequence data (.bcl files) generated by Illumina NovaSeq were converted to fastq files and demultiplexed using Illumina bcl2fastq 2.20. Trimmomatic v.0.36 was then used to remove adapter sequences. The final trimmed reads were mapped to the Oreochromis_niloticus reference genome available on ENSEMBL with STAR aligner v.2.5.2b. Statistics of mapped reads are provided in **Supplementary File 1**. Unique gene counts were further calculated by the function of featureCounts from Subread v.1.5.2 (**Supplementary File 2**). Genes with fewer than 10 counts combined in all libraries were removed for downstream RNA-Seq analysis.

### 2.4 Data analysis of RNA-Seq

**Figure 1:**
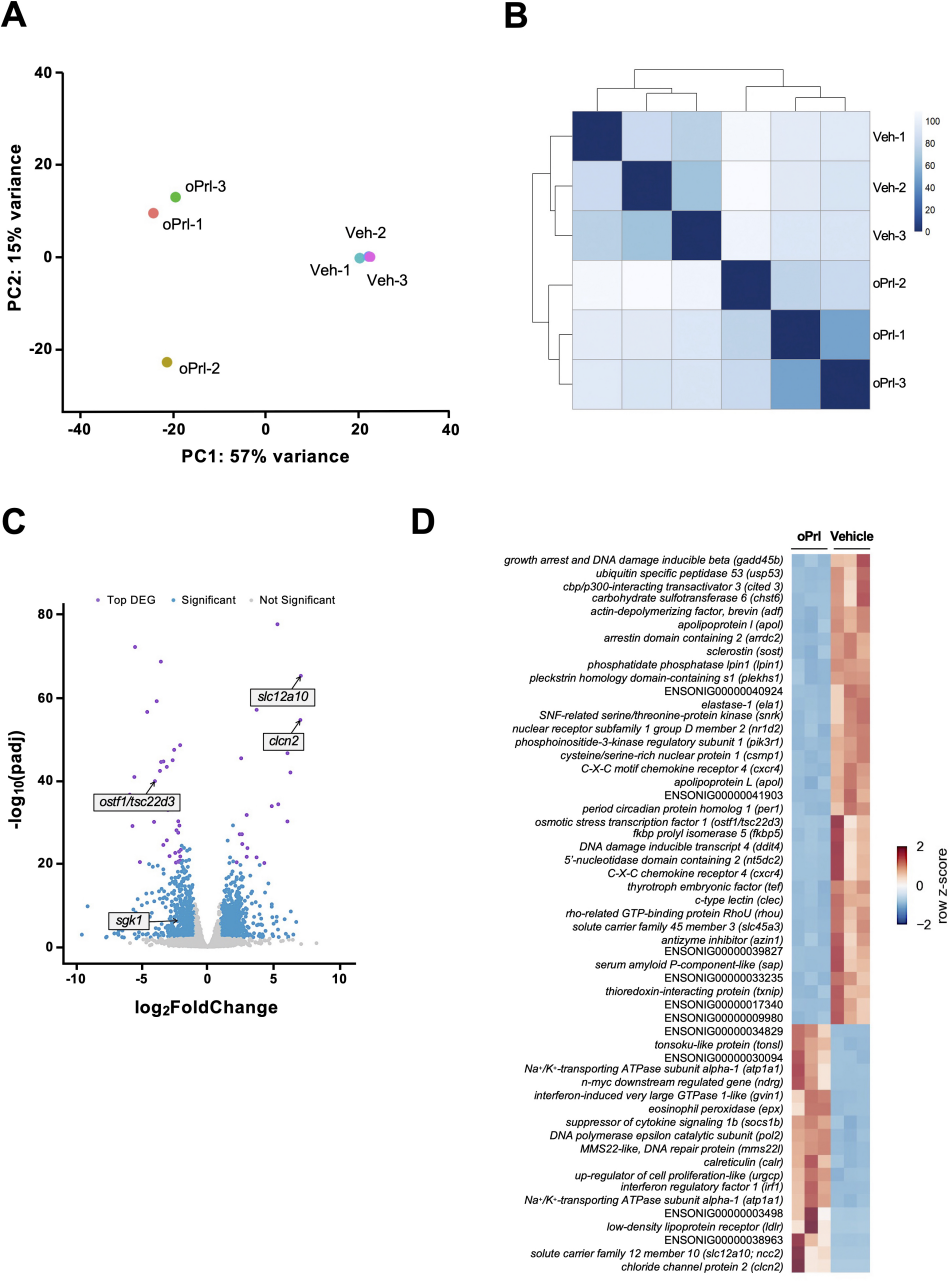
RNA-Seq analysis of hypophysectomized tilapia treated with Prl. A PCA plot showing the similarity between a subset (*n* = 3) of hypophysectomized tilapia injected intraperitoneally with ovine Prl (oPrl; 5 μg/g body weight) or saline vehicle (Veh) **(A)**. Hierarchical clustering of sample-to-sample distance among six RNA-Seq libraries **(B)**. The value in each grid represents the Euclidean distance between two libraries calculated from normalized expression data. Volcano plot of differentially expressed genes (DEGs) identified between the vehicle- and oPrl-injected groups **(C)**. The purple points denote the top DEGs, the blue points denote significant DEGs, and the gray points indicate genes not differentially expressed between groups. Specific genes described in the text are labelled. The horizontal axis represents the average log_2_ (fold change) of the sample and the vertical axis shows −log_10_(padj). See **Supplementary File 4** for the full list of DEGs. Heatmap of relative expression of the top 55 DEGs (i.e., the purple points in the volcano plot) **(D)**. Values in the heatmap represent the z-score of mRNA levels.

DESEQ2 was used to conduct Principal Component Analysis (PCA) and DEG analysis (**Figures 1**). In the PCA, the top 500 variable genes were used to generate a 2D PCA plot. In addition, a correlation analysis was performed to examine similarity among the six RNA-Seq libraries, using Euclidean distance to measure sample-to-sample distance and the Ward method for clustering. Genes with adjusted p-value < 0.01 and |log2 fold change| >1 were selected as DEGs.

### 2.5 Steady-state branchial expression of *cftr, ostf1*, and *sgk1*

To compare the branchial expression of *cftr, ostf1, and sgk1* between tilapia fully acclimated to various environmental salinities, gill filaments were collected from male tilapia held in FW, BW (12‰), or SW for over 1 week (*n* = 5–9).

### 2.6 Western blotting

Gill filaments were ice-sonicated in CelLytic MT Cell Lysis Reagent (Sigma-Aldrich), which contained Halt Protease and Phosphatase Inhibitors Cocktail (ThermoFisher) along with 5 mM EDTA at pH 8.0. Following sonication, gill extracts were centrifuged at 13,000 *g* for 15 min at 4 °C. The supernatant was assessed for protein concentration using Bio-Rad Assay Dye Reagent (Bio-Rad, Hercules, CA) with a bovine serum albumin standard curve. Fifteen µg of total protein extract from gills were diluted 1:1 with 2X Laemmli Sample Buffer (Bio-Rad) containing 100 mM dithiothreitol (Sigma-Aldrich). Samples were boiled for 5 min, then immediately transferred to ice for 5 min before centrifugation. The samples were loaded into a 4-20% SDS-PAGE gel (Criterion TGX Precast Midi Protein Gel; Bio-Rad) flanked by 3 µl of the Precision Plus Protein Dual Xtra Prestained Protein Standard (Bio-Rad) for molecular weight guidance. The gel was run at 150V for 90 min and then assembled into the wet-transfer apparatus (Criterion Blotter with Plate Electrodes) over a methanol-pre-treated PVDF membrane (Immobilon-FL PVDF; Li-Cor, Lincoln, NE) submerged in 1X Tris-Glycine buffer containing 9% methanol. The transfer was conducted for 4 h at 50V. After transfer, the membrane was washed in 1X PBS and incubated in Intercept (TBS) Blocking Buffer (Li-Cor) for 1 h at room temperature. Primary antibody incubations with polyclonal rabbit anti-Sgk (1:1000; S5188, MilliporeSigma, Burlington, MA) and monoclonal mouse anti-β actin (1:1000; 08691001, MPBio, Santa Ana, CA) were carried out overnight at 4 °C. The Sgk antibody, previously used to recognize Sgk1 in *Fundulus heteroclitus* (56), was raised against the C-terminal end peptide (KEAAEAFLGFSYAPPTDSFL) of human Sgk1. According to the ExPASy SIM Alignment tool (57), the corresponding C-terminal sequence of Mozambique tilapia Sgk1 (Uniprot #I6QBE3) shares 80% identity with the Sgk antibody peptide sequence. For a 45-min incubation at room temperature, the infrared secondary antibodies (anti-rabbit IRDye 800CW and anti-mouse IRDye 680RD; Li-Cor) were diluted 1:10,000. After multiple washes with 1X PBS-T and then 1X PBS, the membrane was scanned in an Odyssey CLx Imaging system (Li-Cor) to detect infrared fluorescence. Data were visualized and analyzed using ImageStudioLite software (Li-Cor).

### 2.7 Immunohistochemistry

For whole-mount immunohistochemistry, gill filaments were fixed overnight in 4% paraformaldehyde (PFA) in 0.1 M phosphate-buffered saline (PBS, pH 7.4) at 4 °C. The fixed samples were then washed in PBS containing 0.2% Triton-X 100 (PBST), followed by immersion in a blocking solution (PBST with 2% normal goat serum, 0.1% bovine serum albumin, 0.02% keyhole limpet hemocyanin, and 0.01% sodium azide) overnight at 4 °C. After blocking, the specimens were incubated overnight at room temperature with rabbit anti-Sgk (1:200) and ‘24-1’ monoclonal mouse anti-Cftr (1:500; MAB25031-SP, R&D Systems, Minneapolis, MN) or ‘α5’ monoclonal mouse anti-Nka (1:1000; AB_2166869, Developmental Studies Hybridoma Bank, Iowa City, IA) diluted in blocking solution. Previous use of the ‘24-1’ antibody, raised against the C-terminal region of human Cftr, in tilapia was described by Hiroi et al. (36). The ‘α5’ antibody, raised against the avian Nka α-subunit, is widely used to detect branchial Nka in various teleosts (15). Following several washes with PBST, the filaments were incubated overnight at room temperature with secondary antibodies labeled with fluorescents (donkey anti-rabbit IgG labeled with Alexa Fluor 647 and goat anti-mouse IgG labeled with Alexa Fluor 488, Invitrogen, Waltham, MA) diluted 1:500 in PBST. After rinsing with PBST and PBS, the filaments were mounted on glass slides with 75% glycerol. The specimens were observed using a confocal laser scanning microscope (Olympus Fluoview 1200, Center Valley, PA), and the images were processed with FV10-ASW v4.1 software. Filaments were examined from individuals held in SW for over 2 weeks (*n* = 4).

### 2.8 Effects of salinity transfer on plasma osmolality and branchial *cftr, ostf1*, and *sgk1*

For the SW transfer experiment, eighty FW-acclimated tilapia (~280 g; males) were distributed across ten 700-l tanks supplied with FW. Fish were fed daily to satiation and acclimated to the experimental tanks for > 2 weeks before salinity transfer. On day 0, fish from two tanks were sampled. The water supply to four tanks was then changed to 85% SW (30‰) for 48 h, after which it was adjusted to full-strength SW (FW-SW treatment). Four tanks were maintained as time-matched controls (FW-FW treatment). At sampling (0.25, 1, 2, and 7 d after transfer), fish from one FW-FW tank and one FW-SW tank were quickly netted and lethally anesthetized with 2-PE. Blood plasma and gill filaments were collected and stored as described above.

Eighty tilapia maintained in SW for > 2 years (~360 g; males) were distributed across ten 700-l tanks supplied with SW for the FW transfer experiment. As before, fish were fed daily to satiation and acclimated to the experimental tanks for > 2 weeks before salinity transfer. On day 0, fish from two tanks were sampled. The water supply to four tanks was then switched to FW (SW-FW treatment); FW conditions were reached after 60 min. Four tanks served as time-matched controls (SW-SW treatment). At sampling (0.25, 1, 2, and 7 d after transfer), fish from one SW-SW tank and one SW-FW tank were sampled for plasma and gill filaments. Tilapia in both the SW- and FW-transfer experiments were fasted throughout the trials.

### 2.9 *In vitro* effects of medium osmolality, Prl, and cortisol

After donor fish were lethally anesthetized with 2-PE, gill filaments from the second and third gill arches were removed, and gill filaments were prepared and incubated as previously described (22, 23). Leibovitz’s L-15 culture medium (Gibco/Life Technologies, Carlsbad, CA), supplemented with 6.0 mg/l penicillin and 100 mg/l streptomycin (Sigma-Aldrich), was used in two gill filament incubation experiments. The first experiment assessed the effects of extracellular osmolality by incubating filaments at 280, 330, 380, and 450 mOsmol/kg (*n* = 8). These values reflect plasma osmolality levels that Mozambique tilapia routinely tolerate following changes in environmental salinity (29, 58, 59). The hyposmotic culture medium (280 mOsmol/kg) was produced by diluting Leibovitz’s L-15 culture medium with distilled water. Isosmotic (330 mOsmol/kg) and hyperosmotic (380 and 450 mOsmol/kg) media were produced by adding a 5 mol/l NaCl solution to the hyposmotic medium. The osmolalities of the culture media were verified using a vapor pressure osmometer (Wescor 5520). After adjusting osmolality, all media were sterilized with a 0.2-µm filter. Gill filaments were incubated for 6 h at 26 °C under saturated humidity, with each well (24-well plates; Becton Dickinson, Franklin Lakes, NJ) containing three sagittally cut gill filaments and 500 μl of culture medium. For the second experiment, the individual and combined effects of medium osmolality, oPrl, and cortisol were investigated by incubating filaments for 6 h in either 330 or 450 mOsmol/kg medium supplemented with oPrl (5.0 μg/ml), cortisol (1.0 μg/ml; Sigma-Aldrich), or a combination of oPrl and cortisol (*n* = 8). The concentrations of Prl and cortisol were based on previous studies (22, 60). Both experiments were terminated by collecting the incubated gill filaments in TRI Reagent before their storage at -80 °C.

### 2.10 RNA extraction, cDNA synthesis, and quantitative real-time PCR

Total RNA was extracted from homogenized tissues using the TRI Reagent procedure. RNA concentration and purity were assessed by spectrophotometric absorbance (NanoDrop One). First-strand cDNA was synthesized by reverse transcribing 50–100 ng of total RNA with a High Capacity cDNA Reverse Transcription Kit (Life Technologies). Relative levels of mRNA were determined by qRT-PCR using the StepOnePlus real-time PCR system (Life Technologies). Primers for *sgk1* (XM_005449566.4) were designed using NCBI Primer-BLAST: F: GAGGAGTCTCCGGACAACAA and R: GGATGACGGGCAGACTTTAT (product = 96 bp). The reverse primer spans a predicted exon-exon junction. Melt curve analysis was performed to assess non-specific product amplification and primer-dimer formation. The amplification efficiency of the *sgk1* primers was 97%. We employed previously validated primer sets for *cftr* (28), *clc2c* (24), *ncc2* (61), *prlr1* (27), *prlr2* (20), and *ostf1* (62). **Supplementary File 3** provides the primer sets used to validate additional DEGs. *Elongation factor 1α* levels were used to normalize target gene expression (63). qRT-PCR reactions were set up and cycled as described by Inokuchi et al. (22). Reference and target genes were calculated by the relative quantification method with PCR efficiency correction (64). Standard curves were prepared from serial dilutions of gill cDNA and included on each plate to calculate the PCR efficiencies for target and reference gene assays. Gene expression differences between groups are reported as fold changes relative to controls.

### 2.11 Statistical analyses

**Figure 2:**
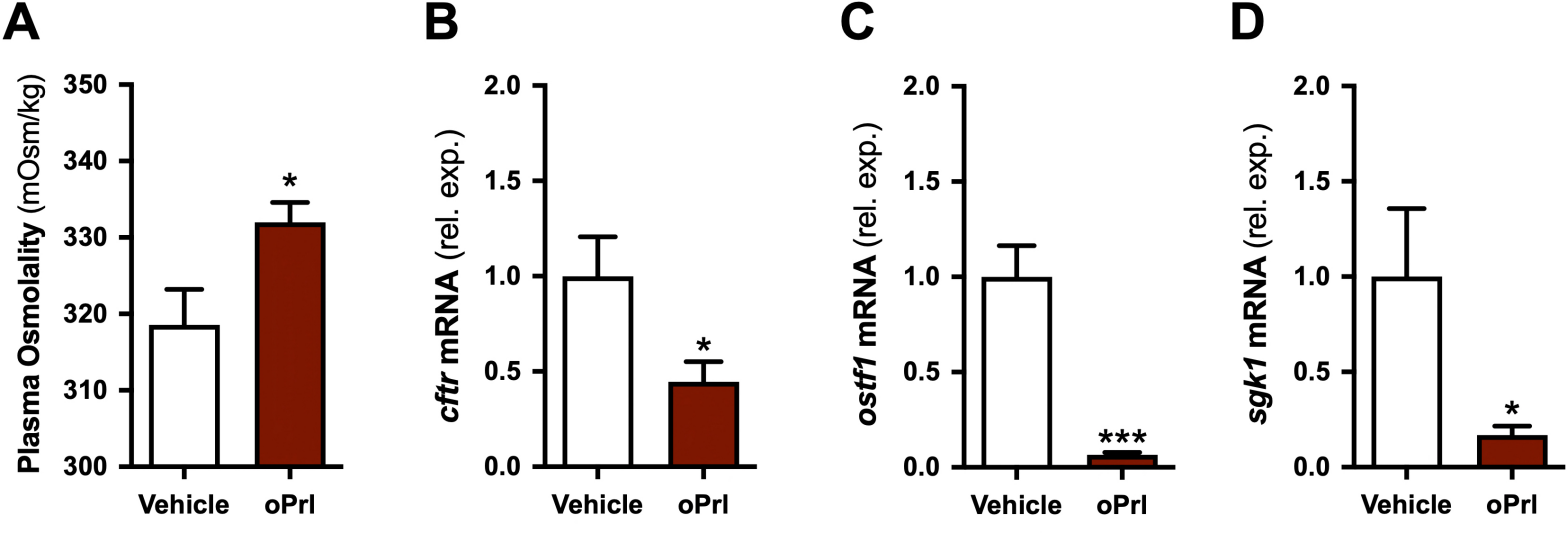
Effect of Prl on selected gene transcripts in hypophysectomized tilapia. Plasma osmolality **(A)** and branchial mRNA levels of *cftr* **(B)**, *ostf1* **(C)**, and *sgk1* **(D)** in hypophysectomized tilapia injected with oPrl (solid red bars) or saline vehicle (open bars). Means ± S.E.M. (*n* = 8). mRNA levels are presented as fold changes relative to the vehicle-injected controls. Group differences were analyzed by Student’s *t* test. *P < 0.05 and ***P < 0.001.

**Figure 3:**
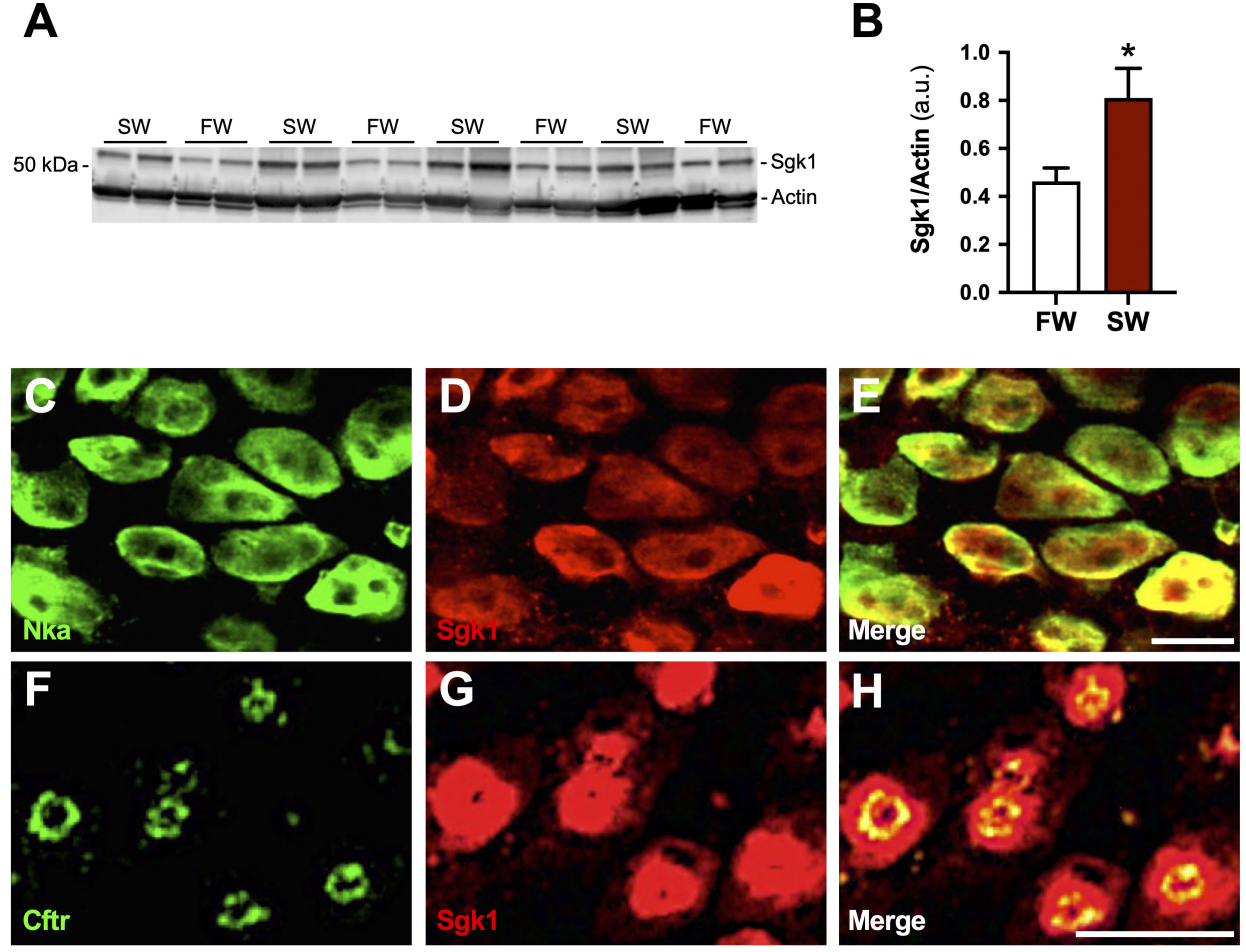
Sgk1 expression in ‘seawater-type’ ionocytes. Sgk1 and β-actin protein abundance in gill filaments collected from freshwater (FW)- and seawater (SW)-acclimated tilapia assessed by Western blot analysis **(A)**. Sgk1 expression was normalized to β-actin and analyzed by Student’s *t* test (*P < 0.05). Means ± S.E.M. (*n* = 8) **(B)**. Double immunofluorescent labeling of Nka (green) **(C)** and Sgk1 (red) **(D)** in the afferent-vascular edge of gill filaments of SW-acclimated tilapia. Merged image of Nka and Sgk1 immunofluorescence showing Sgk1 immunoreactivity within Nka-expressing ionocytes **(E)**. Scale bar = 20 μm. Double immunofluorescent labeling of Cftr (green) **(F)** and Sgk1 (red) **(G)** in gill filaments of SW-acclimated tilapia. Merged image of Cftr and Sgk1 immunofluorescence showing Sgk1 immunoreactivity within Cftr-expressing ionocytes **(H)**. Scale bar = 40 μm.

**Figure 4:**
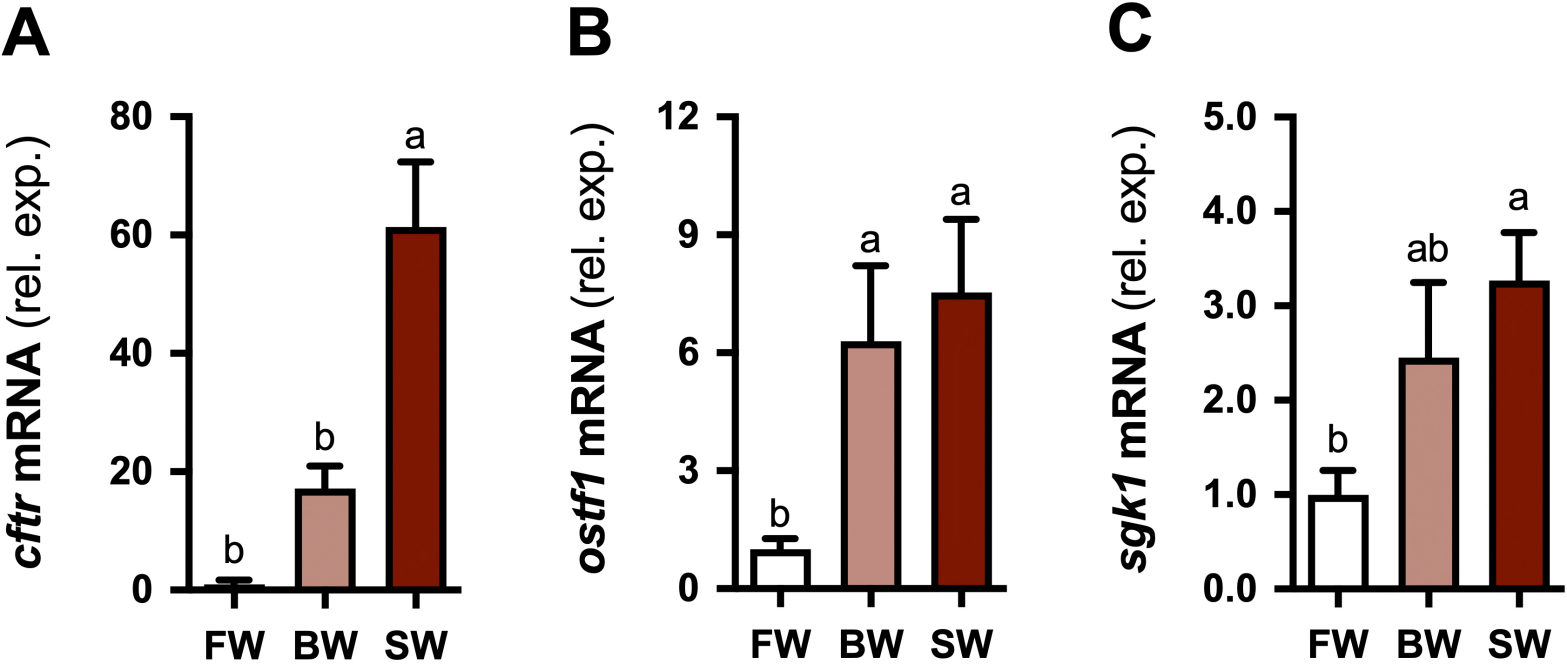
Effect of environmental salinity on steady-state branchial gene expression. Branchial *cftr* **(A)**, *ostf1* **(B)**, and *sgk1* **(C)** mRNA levels in freshwater (FW)-, brackish water (BW; 12‰)-, and seawater (SW)-acclimated tilapia. Means ± S.E.M. (*n* = 5-9). mRNA levels in BW (shaded red bars) and SW (solid red bars) are presented as fold changes relative to FW (open bars). Means that do not share the same letter are significantly different (one-way ANOVA, Tukey’s HSD test, *P* < 0.05).

**Figure 5:**
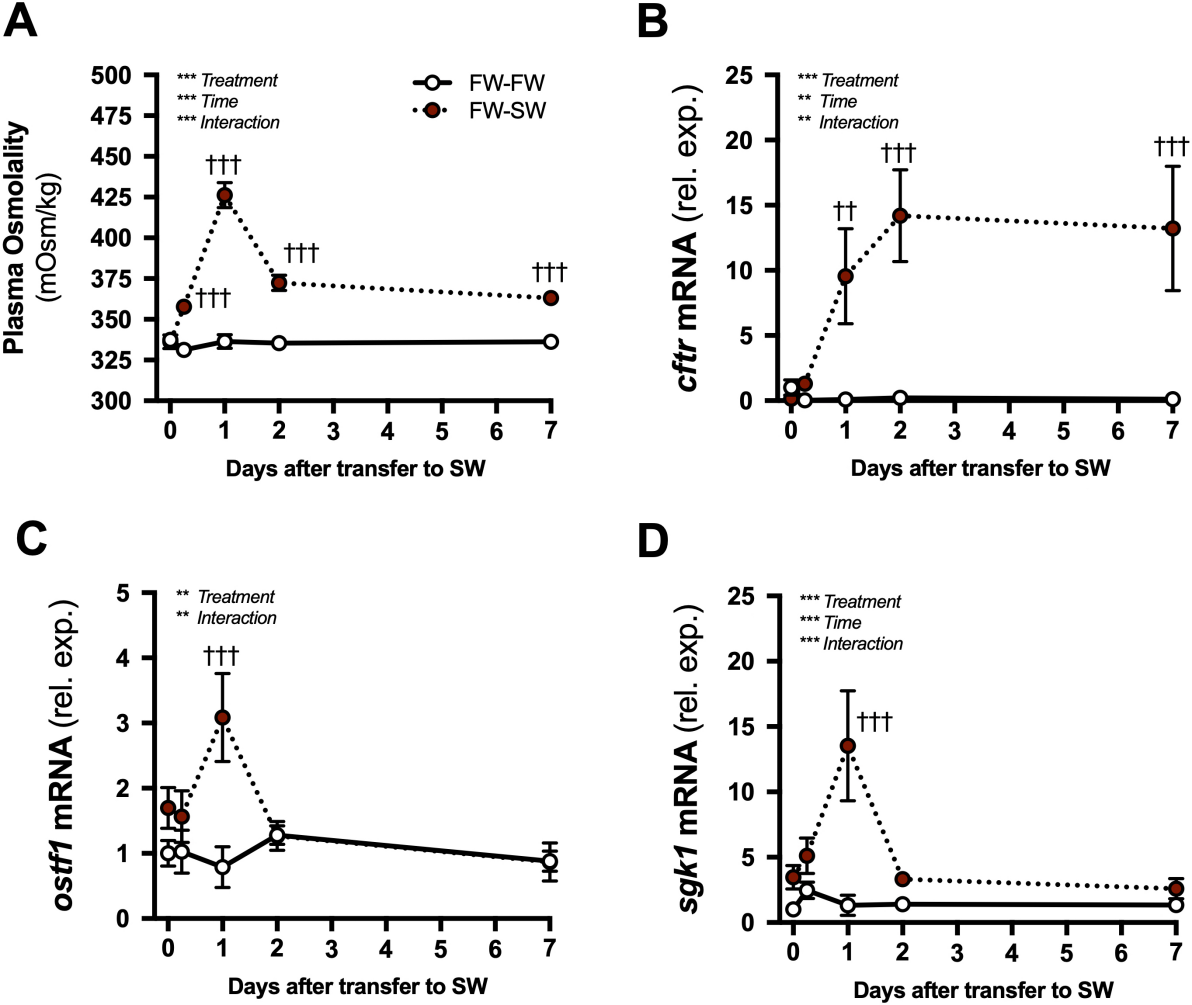
Time courses of branchial gene expression responses to seawater transfer. Plasma osmolality **(A)**, and branchial *cftr* **(B)**, *ostf1* **(C)**, and *sgk1* **(D)** mRNA levels after transfer of tilapia from fresh water (FW) to seawater (SW; solid red symbols with dotted line). Time-matched control fish were maintained in FW (open symbols with solid line). Means ± S.E.M. (*n* = 7-8). mRNA levels are presented as fold changes relative to the FW-FW group at time 0. Differences among groups were evaluated by two-way ANOVA. Significant effects of treatment, time, or an interaction are indicated in respective panels (**P < 0.01 and ***P < 0.001). When there was a significant treatment or interaction effect, *post hoc* comparisons (Bonferroni’s multiple comparisons test) were made at each time point (^††^*P* < 0.01 and ^†††^*P* < 0.001).

**Figure 6:**
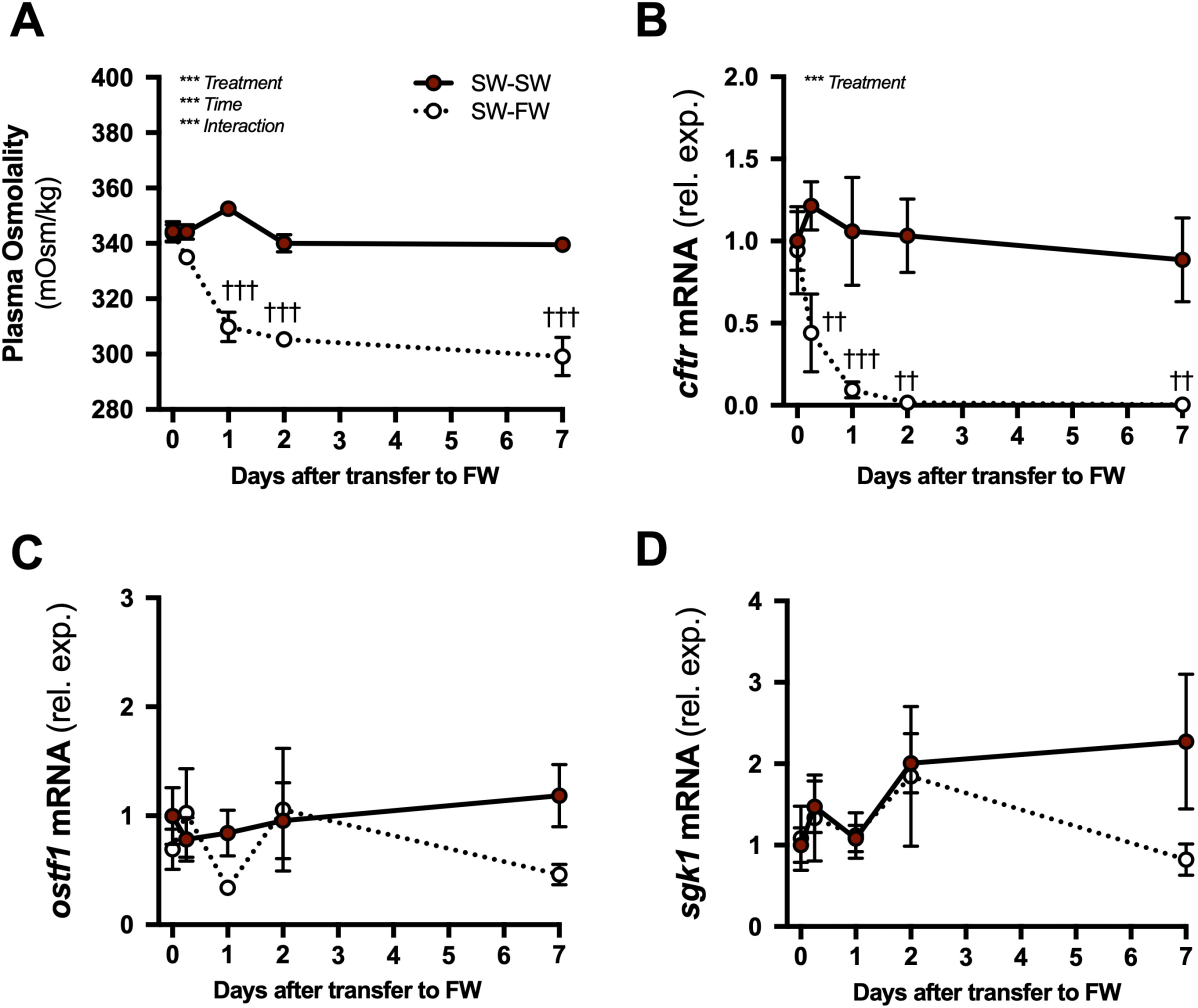
Time courses of branchial gene expression responses to freshwater transfer. Plasma osmolality **(A)**, and branchial *cftr* **(B)**, *ostf1* **(C)**, and *sgk1* **(D)** mRNA levels after transfer of tilapia from seawater (SW) to fresh water (FW; open symbols with dotted line). Time-matched control fish were maintained in SW (solid red symbols with solid line). Means ± S.E.M. (*n* = 8). mRNA levels are presented as fold changes relative to the SW-SW group at time 0. Differences among groups were evaluated by two-way ANOVA. Significant effects of treatment, time, or an interaction are indicated in respective panels (***P < 0.001). When there was a significant treatment or interaction effect, *post hoc* comparisons (Bonferroni’s multiple comparisons test) were made at each time point (^††^*P* < 0.01 and ^†††^*P* < 0.001).

**Figure 7:**
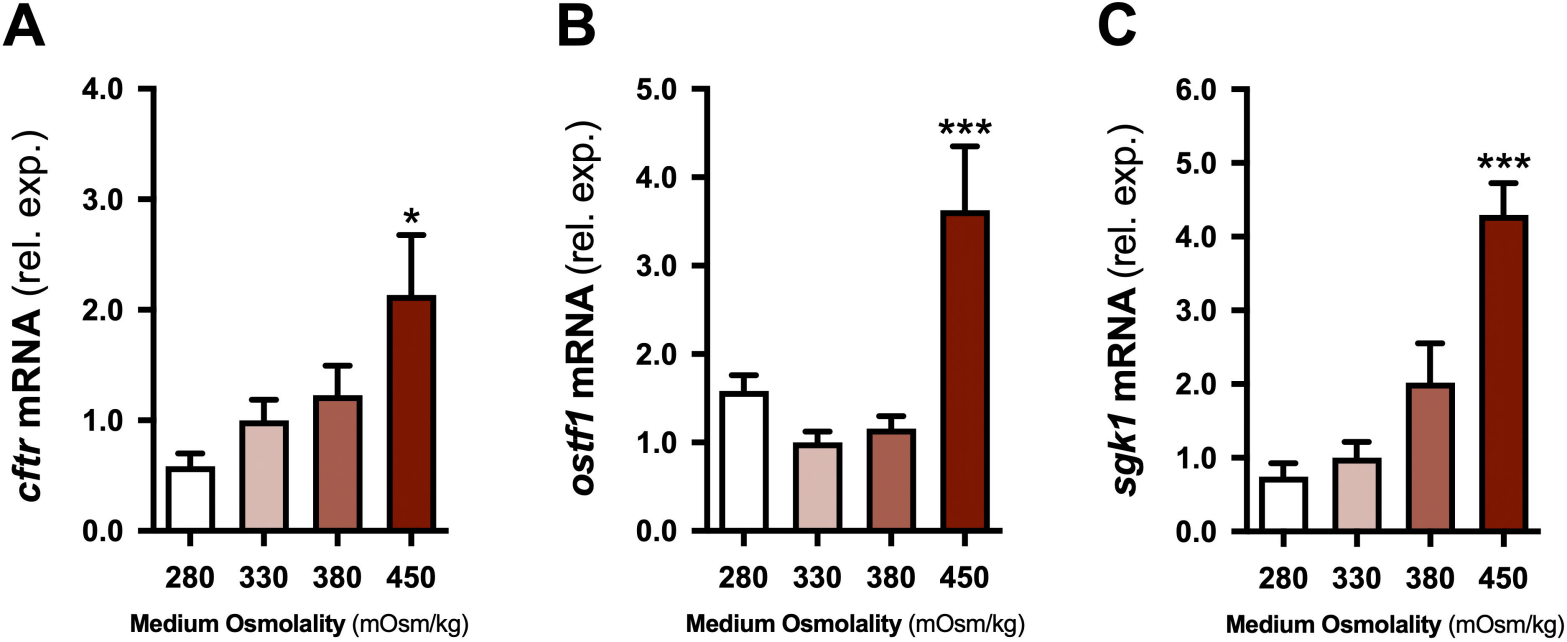
Effects of medium osmolality on *cftr*, *ostf1*, and *sgk1* in incubated gill filaments. Branchial *cftr* **(A)**, *ostf1* **(B)**, and *sgk1* **(C)** mRNA levels in filaments incubated for 6 h in conditions ranging from 280 to 450 mOsm/kg. Means ± S.E.M. (*n* = 8). mRNA levels are presented as fold changes relative to the 330 mOsm/kg (control) group. Asterisks indicate a significant difference from the control group (one-way ANOVA; Dunnett’s test; *P < 0.05 and ***P < 0.001).

**Figure 8:**
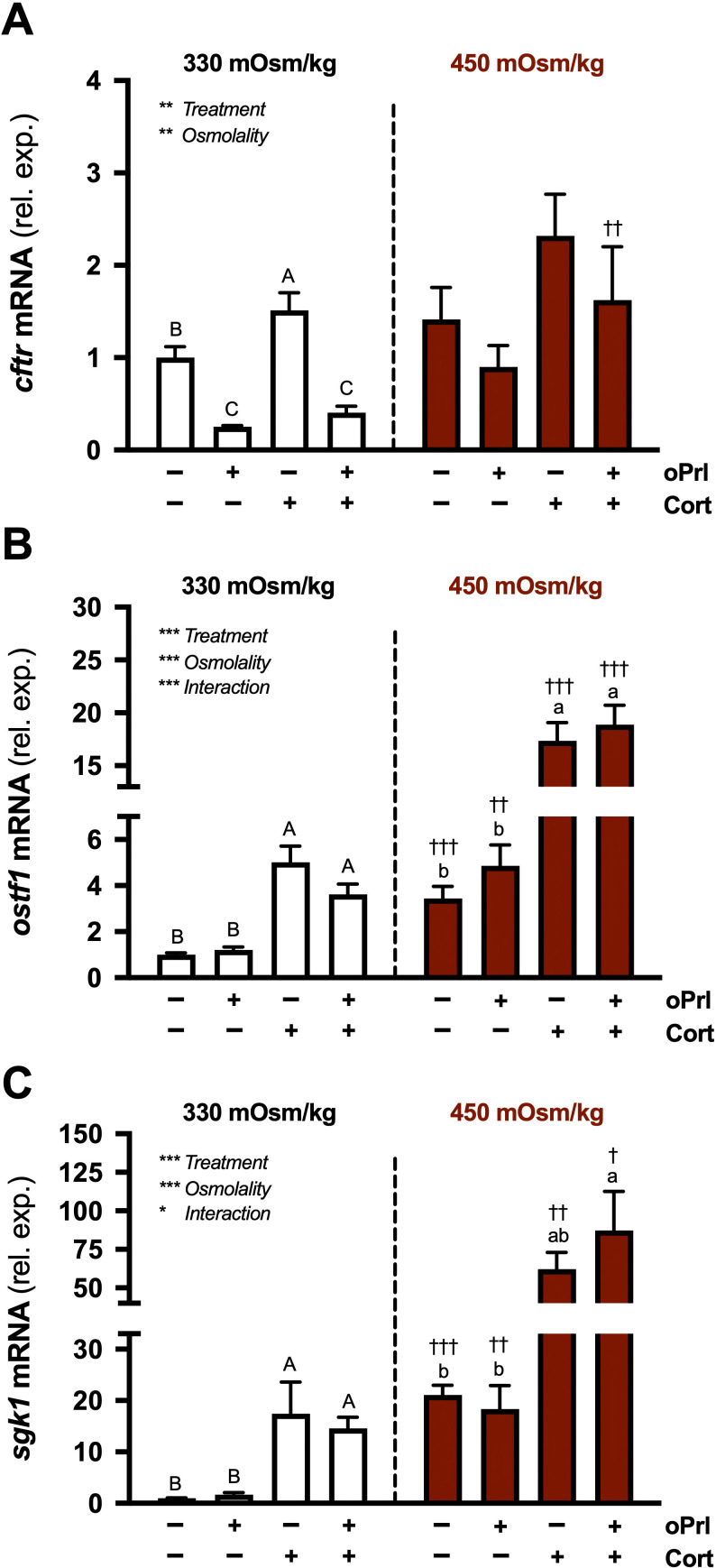
Effects of osmolality, Prl, and cortisol on *cftr*, *ostf1*, and *sgk1* in incubated gill filaments. Branchial *cftr* **(A)**, *ostf1* **(B)**, and *sgk1* **(C)** mRNA levels in filaments incubated for 6 h at either 330 or 450 mOsm/kg in the presence of ovine Prl (oPrl; 5 μg/ml) or cortisol (Cort; 1 μg/ml) alone or in combination. Means ± S.E.M. (*n* = 8). mRNA levels are presented as fold changes relative to the untreated group at 330 mOsm/kg. Differences among groups were evaluated by two-way ANOVA. Significant effects of treatment, osmolality, or an interaction are indicated in respective panels (*P < 0.05, **P < 0.01, and ***P < 0.001). When there was a significant treatment effect, *post hoc* comparisons (one-way ANOVA, Tukey’s HSD test, *P* < 0.05) were made between treatments within each osmolality. For a given osmolality, denoted by uppercase or lowercase letters, means that do not share the same letter are significantly different. When there was a significant osmolality effect, *post hoc* comparisons (Bonferroni’s multiple comparisons test) were made between treatment-matched groups (^†^*P* < 0.05, ^††^*P* < 0.01, and ^†††^*P* < 0.001).

**Figure 9:**
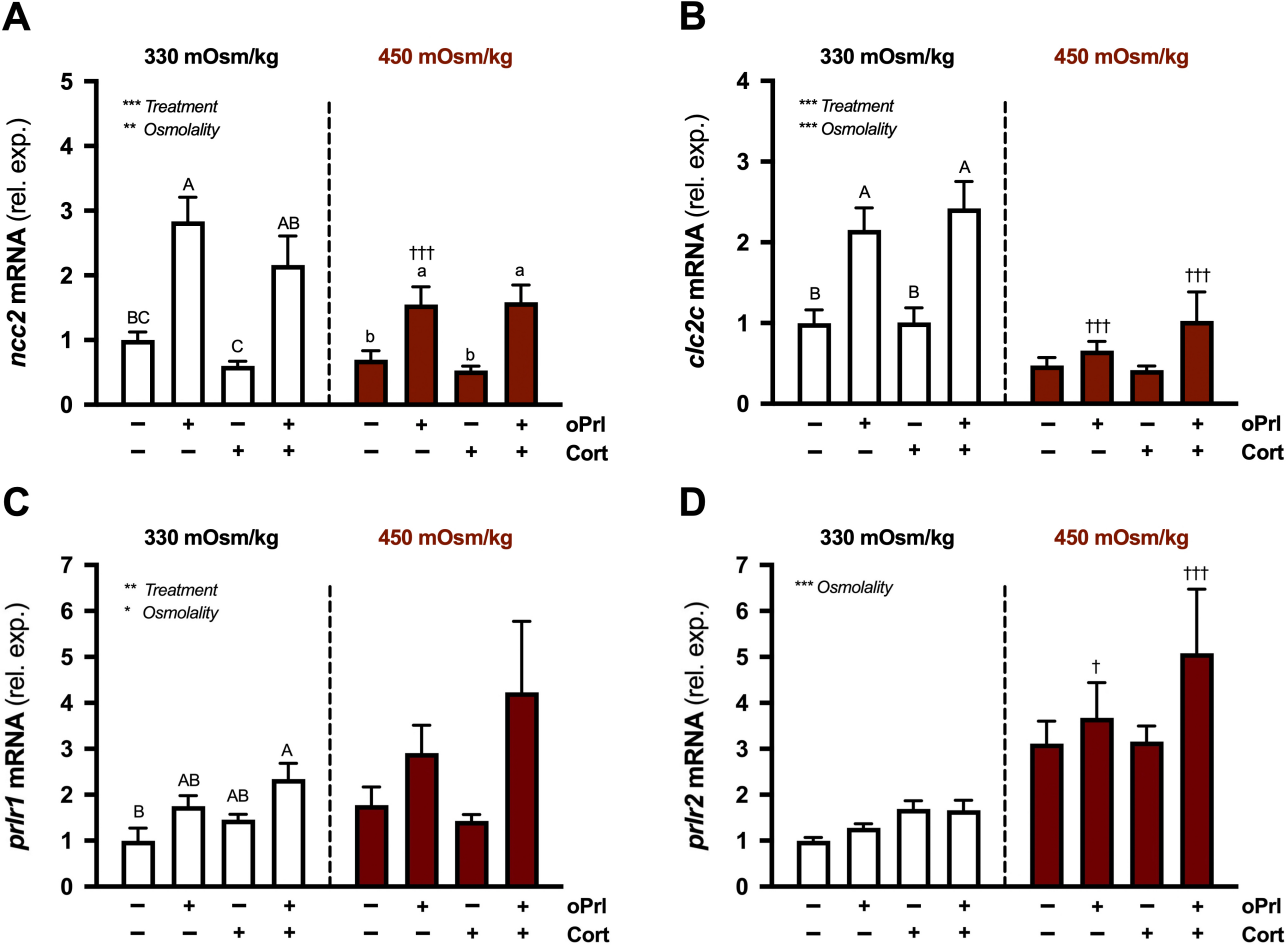
Effects of osmolality, prolactin, and cortisol on *ncc2*, *clc2c*, *prlr1*, and *prlr2* in incubated gill filaments. Branchial *ncc2* **(A)**, *clc2c* **(B)**, *prlr1* **(C)**, and *prlr2* **(D)** mRNA levels in filaments incubated for 6 h at either 330 or 450 mOsm/kg in the presence of ovine prolactin (oPrl; 5 μg/ml) or cortisol (Cort; 1 μg/ml) alone or in combination. Means ± S.E.M. (*n* = 8). mRNA levels are presented as fold changes relative to the untreated group at 330 mOsm/kg. Differences among groups were evaluated by two-way ANOVA. Significant effects of treatment or osmolality are indicated in respective panels (*P < 0.05, **P < 0.01, and ***P < 0.001). When there was a significant treatment effect, *post hoc* comparisons (one-way ANOVA, Tukey’s HSD test, *P* < 0.05) were made between treatments within each osmolality. For a given osmolality, denoted by uppercase or lowercase letters, means that do not share the same letter are significantly different. When there was a significant osmolality effect, *post hoc* comparisons (Bonferroni’s multiple comparisons test) were made between treatment-matched groups (^†^*P* < 0.05 and ^†††^*P* < 0.001).

For single comparisons, differences between groups were analyzed using Student’s *t* test (**Figures 2**, **3B**). Multiple group comparisons were conducted by one-way ANOVA followed by Tukey’s HSD test (**Figure 4**). The salinity transfer experiments (**Figures 5**, **6**) were evaluated by two-way ANOVA. When significant treatment or interaction effects were detected, *post hoc* comparisons (Bonferroni’s multiple comparisons test) were made between time-matched groups. In the first *in vitro* experiment (**Figure 7**), group comparisons were conducted by one-way ANOVA followed by Dunnett’s test. The second *in vitro* experiment (**Figures 8**, **9**) was analyzed using two-way ANOVA. When a significant treatment effect was detected, *post hoc* comparisons (one-way ANOVA, Tukey’s HSD test) were performed within each osmolality. When significant osmolality or interaction effects were detected, *post hoc* comparisons (Bonferroni’s multiple comparisons test) were made between treatment-matched groups. All statistical analyses were performed using GraphPad Prism 10 (GraphPad Software, San Diego, CA). The significance level for all tests was set at *P* < 0.05.

## 3 Results

### 3.1 Prl affects branchial gene expression in hypophysectomized tilapia

To explore transcriptomic profiles of hypophysectomized tilapia injected with either saline or oPrl, we performed RNA-Seq on a subset of individuals from the hypophysectomy/replacement experiment. All raw sequencing data generated in this study have been deposited in NCBI’s Gene Expression Omnibus (Acc. No. GSE324306). Results of PCA and correlation analysis from RNA-Seq not only showed decent sample-to-sample similarity within each condition but also revealed global transcriptomic differences between vehicle and oPrl-injected samples (**Figures 1A, B**). In total, we identified 1,456 DEGs as shown in the volcano plot (**Figure 1C****;** **Supplementary File 4**).

We first observed that two genes previously established as targets of Prl signaling (20, 24), namely *slc12a10* (*ncc2*) and *clcn2* (*clc2c*), were upregulated by oPrl (**Figure 1C**). In contrast, our analysis revealed that *ostf1* and *sgk1* were downregulated by oPrl. While examining the top DEGs (adjusted p-value < 1e-20 and |log2 fold change| > 2), we identified genes that may play important roles in associating Prl signaling with salinity acclimation (**Figure 1D**). Among the Prl-repressed genes, we identified a H^+^-dependent sugar transporter (*slc45a3*), a glucocorticoid receptor co-chaperone (*fkbp5*), cellular stress markers (*txnip*, *ddit4*, and *gadd45b*), and cell-cycle regulators (*azin1*, *rhou*, *cxcr4*, and *nt5dc2*). *ostf1* was included in the top DEGs. The top DEGs upregulated by Prl included a Nka subunit (*atp1a1/nka-α1*), a suppressor of cytokine signaling (*socs1b*), cell-cycle regulators (*urgcp* and *ndrg*), a regulator of intracellular Ca^2+^ (*calr*), and immune factors (*irf1* and *gvin1*). *slc12a10/ncc2* and *clcn2/clc2c* were included in the top DEGs.

Next, we analyzed the complete hypophysectomy/replacement experiment to assess whether Prl affects *cftr* and to confirm that Prl represses *ostf1* and *sgk1*, as identified in the RNA-Seq analysis. This was warranted because only three samples per treatment were used in the initial RNA-Seq experiment, which may have affected overall false-positive rates and statistical power. As reported previously (24), plasma osmolality was higher in hypophysectomized fish that received oPrl compared to vehicle injections (**Figure 2A**). Tilapia injected with oPrl exhibited *cftr*, *ostf1*, and *sgk1* levels that were 0.5-, 0.1-, and 0.2-fold those observed in vehicle-injected controls (**Figures 2B–D**). Eight DEGs (*atf6*, *ccnb3*, *cks1b*, *dna2*, *kif11*, *kif20a*, *kiff22*, and *ncapd2*) involved in cell-cycle regulation, which were identified in our initial RNA-Seq analysis, were confirmed to be similarly upregulated by Prl in the complete hypophysectomy/replacement experiment (**Supplementary File 5**). With *cftr*, *ostf1*, and *sgk1* identified as Prl-repressed genes, our subsequent experiments examined their regulation by salinity, osmotic conditions, and hormones.

### 3.2 Expression of *cftr, ostf1*, and *sgk1* is sensitive to steady-state salinity conditions

Branchial *cftr* levels were higher in long-term SW-acclimated tilapia compared to FW- and BW-acclimated tilapia (**Figure 4A**). *ostf1* levels were elevated in BW- and SW-acclimated tilapia relative to FW-acclimated tilapia (**Figure 4B**). *sgk1* levels were higher in SW-acclimated tilapia compared to FW-acclimated tilapia; *sgk1* levels in BW did not differ from those in either FW or SW (**Figure 4C**).

### 3.3 Sgk1 protein is expressed in ‘SW-type’ ionocytes

Western blots probed with the Sgk antibody revealed an immunoreactive band with an apparent molecular mass of ~50 kDa (**Figure 3A**). This mass approximates the mass reported for Sgk1 in mummichogs by Shaw et al. (56). Quantification revealed significantly higher Sgk1 protein levels in SW- versus FW-acclimated tilapia (**Figure 3B**). Sgk1-immunoreactivity was detected within Nka- and Cftr-positive ionocytes of gill filaments in SW-acclimated tilapia (**Figures 3C–H**). Sgk1-immunoreactivity showed a cytosolic pattern, in contrast to the apical localization of Cftr.

### 3.4 Salinity transfers impact plasma osmolality and branchial *cftr, ostf1*, and *sgk1* expression

In the FW to SW transfer, there were significant main effects of treatment and time, along with an interaction, on plasma osmolality (**Figure 5A**). Plasma osmolality increased at 0.25, 1, 2, and 7 d after transfer to SW compared to FW-maintained controls. Similarly, significant main effects of treatment and time, and an interaction, affected *cftr* levels (**Figure 5B**). *cftr* levels were elevated above controls at 1, 2, and 7 d after transfer from FW to SW. There was a significant main effect of treatment and an interaction with time on *ostf1* and *sgk1* levels; there was also a significant main effect of time on *sgk1* (**Figures 5C, D**). *ostf1* and *sgk1* were elevated above controls at 1 d after transfer from FW to SW.

In the SW to FW transfer, there were significant main effects of treatment and time, in addition to an interaction, on plasma osmolality (**Figure 6A**). Compared to SW-maintained controls, plasma osmolality was lower at 1, 2, and 7 d after transfer from SW to FW. Treatment significantly affected *cftr* (**Figure 6B**). Relative to controls, *cftr* levels decreased at 0.25, 1, 2, and 7 d after transfer from SW to FW. No significant main or interaction effects were observed on *ostf1* and *sgk1* levels following transfer from SW to FW (**Figures 6C, D**).

### 3.5 Hyperosmolality stimulates *cftr, ostf1*, and *sgk1* expression

To assess the direct effects of extracellular osmolality on *cftr*, *ostf1*, and *sgk1* expression levels, isolated gill filaments were incubated under conditions ranging from 280 to 450 mOsm/kg. After 6 h, *cftr*, *ostf1*, and *sgk1* levels in the 450 mOsm/kg group were 2.1-, 3.6-, and 4.3-fold those observed in the isosmotic (330 mOsm/kg) control group, respectively (**Figure 7**).

### 3.6 Osmolality, Prl, and cortisol interact to regulate *cftr, ostf1*, and *sgk1* expression

There were significant main effects of treatment and osmolality on *cftr* expression (**Figure 8A**). In filaments incubated at 330 mOsm/kg, oPrl, whether alone or in combination with cortisol, reduced *cftr* levels; conversely, cortisol modestly stimulated *cftr* compared to controls. No significant differences in *cftr* were observed between treatments incubated at 450 mOsm/kg. There were significant main effects of both treatment and osmolality, along with an interaction on *ostf1* expression (**Figure 8B**). Regardless of medium osmolality, cortisol, whether alone or in combination with oPrl, stimulated *ostf1*. oPrl alone did not influence *ostf1* levels in filaments incubated at either 330 or 450 mOsm/kg. Like *ostf1*, there were significant main effects of treatment and osmolality, as well as an interaction on *sgk1* expression (**Figure 8C**). At 330 mOsm/kg, cortisol, whether alone or in combination with oPrl, stimulated *sgk1*. In comparison, at 450 mOsm/kg, only cortisol, in the presence of oPrl, elevated *sgk1* compared to controls.

We confirmed that our gill incubation system accurately models *in vivo* responses to Prl by demonstrating that oPrl stimulated *ncc2* and *clc2c* (**Figures 9A, B**) (20, 22, 24). For both *ncc2* and *clc2c*, we observed significant main effects of treatment and osmolality. oPrl alone stimulated *ncc2* in filaments incubated at 330 and 450 mOsm/kg (**Figure 9A**). oPrl stimulated *clc2c* expression in filaments incubated at 330 mOsm/kg, but not in filaments at 450 mOsm/kg (**Figure 9B**). The effectiveness of our *in vitro* system was also indicated by cortisol’s stimulation of *ostf1* (**Figure 8B**) (65).

There were significant main effects of treatment and osmolality on *prlr1* expression (**Figure 9C**). In filaments incubated at 330 mOsm/kg, oPrl in combination with cortisol stimulated *prlr1* levels relative to controls. No significant differences in *prlr1* were observed between treatments at 450 mOsm/kg. For *prlr2*, there was a significant effect of medium osmolality. In groups treated with oPrl, either alone or in combination with cortisol, *prlr2* levels were elevated at 450 mOsm/kg compared with 350 mOsm/kg (**Figure 9D**).

## 4 Discussion

Using Mozambique tilapia as a model, Foskett and Scheffey (66) provided the first direct evidence that teleost ionocytes actively secrete Cl^-^. In the succeeding decades, the molecular workings of ‘SW-type’ ionocytes were uncovered, enabling the establishment of mechanistic links between hormones, cell-autonomous osmosensory processes, and the solute transporters/channels responsible for ion secretion (18, 38). However, a knowledge gap remains because the endocrine and osmosensory pathways that activate and recruit ‘SW-type’ ionocytes are much better understood than those that inhibit ion secretion when euryhaline species enter FW. To address this gap, we first identified a set of genes sensitive to Prl signaling by combining transcriptomic analyses with the ‘classic’ experimental paradigm of hypophysectomy and hormone replacement. We then narrowed our focus to Prl’s inhibition of ion-secretory pathways by examining three transcripts more closely to assess their regulation. Our primary findings include: Prl’s capacity to antagonize cortisol-stimulated *cftr* expression depends on extracellular osmotic conditions; Prl and cortisol play opposing roles in regulating branchial *ostf1* expression; and Sgk1 is expressed in tilapia ‘SW-type’ ionocytes, where interactions among Prl, cortisol, and extracellular osmolality influence its expression.

The salinity-dependent expression of *cftr* observed in this study (**Figures 4A**, **5B**, **6B**) aligns with its encoded protein facilitating the apical exit of Cl^-^ from ‘SW-type’ ionocytes (36). The results of our hypophysectomy (**Figure 2B**) and gill incubation experiments (**Figure 8A**), in which Prl reduced *cftr* expression, suggest that Cftr participates in Prl-mediated inhibition of Cl^-^ secretion (67, 68). In other examined euryhaline species, Prl inhibits branchial *cftr* expression in addition to downregulating Na^+^/K^+^-ATPase activity and *nkcc1* expression (25, 69, 70). When tilapia transition from SW to FW, plasma Prl levels and branchial *prlr1* expression increase within 6 h (29); therefore, the activation of Prl signaling that occurs in response to FW aligns temporally with the decline in *cftr* (**Figure 6B**). Longer-term effects follow the *cftr* response to Prl, when Prl suppresses cell morphologies associated with ‘SW-type’ ionocytes (50, 71). Conversely, during SW-acclimation, when circulating Prl levels are reduced (58), *cftr* expression rises markedly (**Figure 5B**). Recall that cortisol stimulated *cftr* in filaments incubated at 330 mOsm/kg (**Figure 8A**), a pattern consistent with its well-established role in promoting the activity of ‘SW-type’ ionocytes (18). A key finding of this study was that cortisol, when co-administered with Prl, did not stimulate *cftr* expression. Thus, under steady-state FW conditions, when extracellular osmolality approximates 330 mOsm/kg and plasma Prl levels are elevated (58), Prl prevents cortisol from maladaptively upregulating *cftr*. We also discovered that when filaments are exposed to hyperosmotic conditions, Prl’s capacity to oppose cortisol is lost. Therefore, the spike in plasma osmolality that occurs when tilapia enter SW may ‘override’ Prl’s antagonistic effect on cortisol-induced *cftr* expression. Cortisol released after an acute exposure to SW (72) is thus ‘permitted’ to promote *cftr* expression.

Although it makes intuitive sense from an adaptive perspective that hyperosmolality suppresses Prl signaling, thereby enabling ‘SW-type’ ionocytes to increase Cl^-^ secretion via Cftr in response to SW exposure, the mechanisms by which osmotic conditions override Prl’s branchial actions remain unknown. We propose that Prlr2, due to its robust expression under hyperosmotic conditions as shown here (**Figure 9D**) and previously (22, 26), redirects Prl signaling away from activities that promote FW acclimation. For instance, Prlr2 activation increases the expression of *suppressor of cytokine signaling* genes (26), which, through their translated products, could attenuate Prlr1-mediated signaling pathways (73). Prlr1 is likely the mediator of Prl’s FW-adaptive effects because low environmental salinities and Prl itself upregulate expression of its associated gene transcript (22, 29). It is also important to recognize that reduced sensitivity to Prl signaling under hyperosmotic conditions coincides with increased sensitivity to cortisol’s effects. For instance, when tilapia transition from FW to SW, the expression of branchial glucocorticoid and mineralocorticoid receptors (*gr2* and *mr*) is upregulated (74). Hence, the various Prl and cortisol receptors, each with distinct osmosensitivities, enable the gill to toggle between prioritizing Prl’s effects versus those of cortisol, depending on environmental osmolality. Mannitol-based *in vitro* experiments are now warranted to further resolve whether *cftr* and *prlr2*, as well as *sgk1*, described below, are differentially responsive to ionic versus non-ionic stress in tilapia.

Since its identification in tilapia (75), Ostf1 has been recognized as a mediator of transcriptional responses to osmotic stress in euryhaline teleosts. For instance, Ostf1 is expressed in the ionocytes of Japanese eel (*Anguilla japonica*) and medaka (*Oryzias latipes*), where it promotes *cftr* expression in response to hyperosmotic extracellular conditions (76–78). *ostf1* transcripts in the tilapia pituitary also respond directly to increases in osmolality (59). Although hyperosmotic induction of its branchial expression can occur without systemic signals, cortisol, the primary mineralocorticoid in teleosts, stimulates *ostf1* (65, 79). Our combined experiments confirmed that branchial *ostf1* is rapidly stimulated by increases in environmental salinity and extracellular osmolality, as well as by cortisol (**Figures 4B**, **5C**, **7B**, **8B**). More notably, our study provides the first evidence of Ostf1’s sensitivity to Prl (**Figures 1C, D**, **2C**), indicating that crosstalk occurs between Prl signaling and hyperosmotic stress pathways. While future investigation is needed to pinpoint the specific signaling pathways linking Prl to Ostf1, functional domains/phosphorylation sites within Ostf1 suggest several potential candidates. These signaling pathways include mitogen-activated protein kinases (MAPKs), cyclin-dependent kinases, and calcium-calmodulin-dependent kinases (75, 78). Our study thus encourages the interrogation of these transduction cascades to determine their sensitivity to Prl. Such investigations are likely to uncover regulatory connections distinct from those linking cortisol, which promotes SW-adaptive phenotypes, to branchial MAPK signaling (80). While Prl showed the capacity to inhibit *ostf1* when administered to tilapia continuously held in BW (**Figure 2C**), *ostf1* can be quickly induced cell-autonomously in tilapia abruptly transferred from FW to SW (75), a process that occurs before the reduction in circulating Prl (58). Therefore, SW/hyperosmolality must provide a sufficiently strong stimulus to overcome Prl’s inhibitory effects and allow an immediate response to SW exposure. In the absence of this osmotic shock, Prl can inhibit *ostf1* expression (**Figure 2C**). We also note that while Prl attenuated *ostf1* expression by over 90% in hypophysectomized tilapia (**Figure 2C**), this effect was not seen in Prl-treated filaments *in vitro* (**Figure 8B**). First, it is plausible that some systemic factor(s) not present in our *in vitro* conditions are necessary for Prl to inhibit *ostf1* (as well as *sgk1*). Considering the role of hormone-sensitive miRNAs (e.g., miRNA-429) in regulating branchial gene transcripts (81), especially *ostf1* (82), this aspect of *ostf1’s* regulation might have been disrupted without the influence of systemic factors beyond Prl and cortisol. We also propose that the substantial difference in the duration of Prl treatment (i.e., 5 days *in vivo* versus 6 hours *in vitro*) may account for the contrasting sensitivity to Prl observed in our study. Future *in vitro* studies will need to employ longer Prl treatment durations to directly address this question.

Vertebrate Sgk1s are regulated by a wide range of stimuli related to cellular stress signaling, including hypertonicity (83, 84). Among the broad spectrum of ion transporters and channels that interact with Sgk1 (directly or indirectly), several are conserved between the ‘SW-type’ ionocytes of fishes and other vertebrate cells specialized for ion transport, including Cftr, Nkcc, and renal outer medullary K^+^ channels (Romks) (5, 84, 85). The sensitivity of branchial *sgk1* to Prl, as revealed in the hypophysectomy paradigm (**Figures 1C**, **2D**), led us to investigate its expression patterns in response to acute salinity changes, during which it emerged as highly responsive to SW exposure. *sgk1* expression spiked after transfer from FW to SW when plasma osmolality reached ~425 mOsm/kg (**Figures 5A, D**), whereas *sgk1* did not respond to FW exposure when plasma osmolality fell below that of SW-maintained controls (**Figures 6A, D**). The responsiveness of *sgk1* to SW, but not FW, aligns with its *in vitro* sensitivity to hyperosmolality (**Figure 7C**). Because Sgk1 is required for the trafficking of Cftr from intracellular vesicles to the apical membrane of mummichog ionocytes (43, 44, 86), we predicted that Sgk1 would be expressed in ‘SW-type’ ionocytes of tilapia. Immunohistochemistry was performed on filaments from SW-acclimated fish given their high Sgk1/*sgk1* expression (**Figures 3A, B**, **4C**); we observed that Nka- and Cftr-positive ionocytes express Sgk1 in a manner consistent with its binding to intracellular proteins that interact with ion channels and transporters (**Figures 3C–H**) (87). Loss-of-function experiments, as leveraged by Notch et al. (43), are now warranted to determine whether Cftr, Nkcc1, Romka, or other ion transporters/channels are regulatory targets of Sgk1 in tilapia. While branchial Sgk1 mediates adaptive responses to SW in mummichogs (43, 86) and, seemingly, in tilapia, the full extent of Sgk1’s deployment across teleost osmoregulatory organs remains to be determined through comparative studies. It will be interesting to assess whether Sgk1 plays a role in the ‘SW-type’ ionocytes of stenohaline marine species, which lack broad plasticity in ion-transport capacity.

Within the landscape of Prl’s myriad actions on vertebrate osmoregulatory systems (88), we define a new role for Prl as an inhibitor of *sgk1* expression (**Figures 1C**, **2D**). Given the connections among Sgk1, Cftr, and Prl in mummichogs and tilapia, Prl’s inhibition of Sgk1 is another facet of how it attenuates branchial processes suited to SW (18). Sgk1’s sensitivity to cortisol was not unexpected given glucocorticoid actions on mammalian Sgk1 (**Figure 8C**) (89); nonetheless, this study is the first to link cortisol to Sgk1 in a teleost. Hypophysectomized tilapia, which lack an intact hypothalamus-pituitary-interrenal axis to activate cortisol secretion, can nevertheless recruit ‘SW-type’ ionocytes in response to hyperosmotic conditions (20). Although perhaps dispensable, cortisol still plays a supporting role in tilapia SW acclimation by stimulating Nka activity, *nka-α1b*, and *nkcc1* expression (23, 80, 90). Thus, cortisol’s effect on *sgk1* and its synergism with hyperosmolality constitute an additional aspect of its SW-adaptive actions in tilapia. Interestingly, the upregulation of *sgk1* expression in mummichogs exposed to SW does not depend on cortisol signaling (86). When glucocorticoid receptors are pharmacologically blocked, hypertonicity is sufficient to increase *sgk1* expression, a pattern that aligns with hypertonic stimulation of Cftr-mediated Cl^-^ secretion in mummichogs (86). Further investigation is required to determine whether cortisol is essential for *sgk1* induction during SW acclimation in tilapia; cortisol may not be necessary given that *sgk1* was induced by hyperosmotic conditions alone (**Figure 7C**). Our hypophysectomy paradigm is particularly well-suited to address this question. As was the case for *ostf1*, *sgk1* showed contrasting sensitivity to Prl *in vivo* versus *in vitro*. Interactions between Sgk1 and Ostf1, as proposed by Wong et al. (91), may underlie the parallel expression of their transcripts across the experimental paradigms used in this study.

In tilapia, Prl is required during FW-acclimation to activate the uptake of environmental Na^+^ and Cl^-^ by ‘FW-type’ ionocytes that express Ncc2 and Clc2c (20, 24). Accordingly, the *in vitro* responses of *ncc2* and *clc2c* to Prl confirm that the filament incubation system used here models Prl’s actions (**Figures 9A, B**) (22–24). We previously examined, separately, the *in vitro* effects of Prl and extracellular osmolality on the expression of ionocyte genes (22). In the present study, our experimental design enabled us to test them in combination; we found that Prl does not stimulate *clc2c* expression under hyperosmotic extracellular conditions (**Figure 9B**). Although this modulation of Prl’s effects by osmolality was not seen for *ncc2* (**Figure 9A**), we hypothesize that it relates to the osmotic sensitivity of *prlr1* and *prlr2* expression, which, through their reciprocal regulatory patterns under hyperosmotic conditions, will attenuate tissue sensitivity to Prl (22, 29). Lastly, we did not observe an effect of cortisol alone or in combination with Prl on *ncc2* or *clc2c* expression. Because Prl-cortisol synergism may occur when gill filaments are incubated for ≥ 24 h (23), we are reluctant to discount a role for cortisol in supporting FW acclimation in tilapia based solely on our findings.

In summary, we combined classic and modern techniques to elucidate further the physiological mechanisms that enable euryhaline fishes to adapt to a wide range of salinities. Tilapia ‘SW-type’ ionocytes are regulated by the combined actions of extracellular osmolality, cortisol, and Prl, particularly in controlling *cftr* and *sgk1* transcript levels, where these signals exhibit synergistic or antagonistic relationships. Future studies are now poised to articulate the functional consequences (e.g., active Cl^-^ secretion) of the various regulatory links we identified to Sgk1. This study also reinforces that Prl plays multiple roles in facilitating FW adaptation, as it not only promotes ion uptake via ‘FW-type’ ionocytes but also suppresses hyperosmotically-induced genes in ‘SW-type’ ionocytes. Ultimately, Mozambique tilapia illustrate how the ionocytes of euryhaline fishes serve as hubs that integrate osmotic stimuli and endogenous hormonal cues to yield phenotypes that adaptively match the environment.

## Data Availability

All raw sequencing data supporting the conclusions of this study have been made publicly available through NCBI’s Gene Expression Omnibus (Acc. No. GSE324306): https://www.ncbi.nlm.nih.gov/geo/query/acc.cgi?acc=GSE324306. Additional contributions presented in the study are included in the supplementary material, further inquiries can be directed to the corresponding author.
