# Supplementary material for: Prolactin, cortisol, and extracellular osmolality regulate cftr, ostf1, and sgk1 in tilapia ionocytes
Source: Front Endocrinol (Lausanne). 2026 Apr 22;17:1802254. doi: 10.3389/fendo.2026.1802254 (PMC13143792; doi:10.3389/fendo.2026.1802254)
Supplement: Supplementary file 3 [file Supplementaryfile3.pdf]

Supplementary Table. Specific primer sequences for quantitative real-time PCR of select DEGs.

Gene	Primer Sequence (5′-3′)	Efficiency (%)	Acc. No.
atf6	F: CCCTGTGATGGATAGTGAGGC<br>R: TCGAACAGGCTTAAGTCCCAC	97	XM_003440029.5
ccnb3	F: CGAGCTGTACCATGAGACCC<br>R: GTGGGCTGTGCTCCTCAAAT	87	XM_005458602.4
cks1b	F: TGATTCACCAACCAGAGCCG<br>R: CCCACATGGCAGTTAGAGCA	107	XM_003452448.5
dna2	F: TGTCGCCATGAACAGGATGA<br>R: TGTGGAATTCGGGAAGACCTTT	107	XM_019359412.2
kif11	F: TGCGTGATATAACGGTGTCCG<br>R: TCGTGTTGAAAGGCCTGCAT	90	XM_003455691.5
kif20a	F: CACCGTTGGTTTTTGGCGAT<br>R: CCATGCCCGTCTATCTGGAA	90	XM_005472109.4
kif22	F: ACATTCTCACATAATATCGCGCA<br>R: CTCTTACACACGGTCCCTCG	98	XM_005463863.4
ncapd2	F: TGCAGCACTGTAAGTCGGTA<br>R: AGACAACCCACTAACCACTTGT	109	XM_005455432.4
