# Supplementary material for: Prolactin, cortisol, and extracellular osmolality regulate cftr, ostf1, and sgk1 in tilapia ionocytes
Source: Front Endocrinol (Lausanne). 2026 Apr 22;17:1802254. doi: 10.3389/fendo.2026.1802254 (PMC13143792; doi:10.3389/fendo.2026.1802254)
Supplement: Supplementary file 5 [file Supplementaryfile5.pdf]

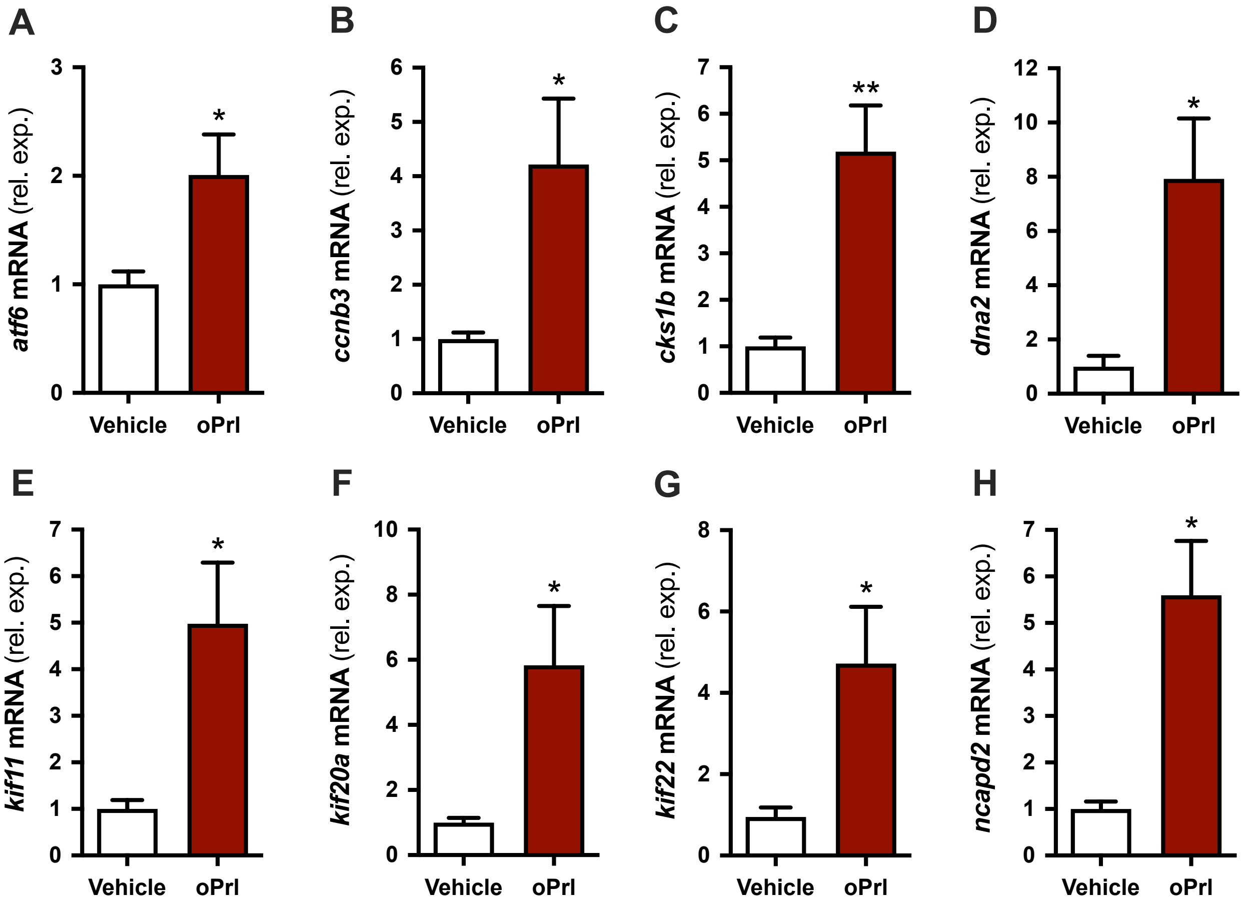

Effect of Prl on the expression of select DEGs in hypophysectomized tilapia. Branchial mRNA levels of *atf6* (A), *ccnb3* (B), *cks1b* (C), *dna2* (D), *kif11* (E), *kif20a* (F), *kiff22* (G), and *ncapd2* (H) in hypophysectomized tilapia injected with oPrl (solid red bars) or saline vehicle (open bars). Means ± S.E.M. (n = 8). mRNA levels are presented as fold changes relative to the vehicle-injected controls. Group differences were analyzed by Student's t test. *P < 0.05 and **P < 0.01.
